# Prevenção da demência com abordagens multimodais

**DOI:** 10.1002/alz70858_102351

**Published:** 2025-12-25

**Authors:** Juliana de Oliveira Alves

**Affiliations:** ^1^ Universidade Feevale, Novo Hamburgo, Rio Grande do Sul, Brazil

## Abstract

**Background:**

Dementia, including Alzheimer's Disease (AD), is a growing public health challenge with significant impacts on individuals and healthcare systems (CRIVELI, 2023). Prevention through multimodal strategies is essential to mitigate this epidemic (ROSENBER, 2020). The complexity of dementia requires approaches that simultaneously target multiple modifiable risk factors. Recent studies have focused on interventions combining lifestyle changes with the potential use of pharmacological agents, such as metformin (BARBERA, 2024).

**Methods:**

A comparative analysis was conducted using three scientific articles from the Medline database, which address methods for dementia prevention.

**Results:**

**MET‐FINGER**: Evaluates the combination of the FINGER 2.0 multimodal intervention with metformin in adults at risk of dementia. Primary outcomes focus on changes in global cognition assessed through a battery of neuropsychological tests.

**LatAm‐FINGERS**: Adapts the FINGER model for Latin America, focusing on lifestyle interventions. Participants are evaluated at multiple time points using culturally adapted neuropsychological tests and undergo biological sample collection and neuroimaging. Both studies include interventions such as healthy diet, regular physical activity, cognitive training, and management of vascular and metabolic risk factors. The WW‐FINGERS network fosters international collaboration among these studies, generating robust and comparable evidence. These studies have demonstrated the feasibility of implementing multimodal interventions in diverse populations with cultural and regional adaptations. The original FINGER study showed significant benefits in cognitive function. Preliminary results from other trials suggest that multimodal interventions may reduce the risk of cognitive decline in high‐risk individuals. The LatAm‐FINGERS study identified a high prevalence of metabolic syndrome, emphasizing the need to address cardiovascular risk factors in dementia prevention.

**Conclusions:**

Multimodal approaches represent a promising strategy for dementia prevention. Combining lifestyle changes with pharmacological interventions may be more effective in addressing the complexity of dementia. Personalizing interventions is crucial to ensure adherence and effectiveness across different cultural and social contexts. International collaboration, through networks such as WW‐FINGERS, is essential to generate solid and comparable evidence. These studies highlight the importance of a global effort to implement dementia prevention strategies and reduce the impact of this condition.

